# An Ecological Momentary Assessment and Intervention Tool for Memory in Chronic Traumatic Brain Injury: Development and Usability of Memory Ecological Momentary Intervention

**DOI:** 10.2196/59630

**Published:** 2024-11-26

**Authors:** Emily L Morrow, Lyndsay A Nelson, Melissa C Duff, Lindsay S Mayberry

**Affiliations:** 1 Department of Medicine, Division of General Internal Medicine and Public Health, Vanderbilt University Medical Center, 2525 West End Avenue, Nashville, TN, 37203, United States; 2 Center for Health Behavior and Health Education, Vanderbilt University Medical Center, Nashville, TN, United States; 3 Department of Hearing & Speech Sciences, Vanderbilt University Medical Center, Nashville, TN, United States

**Keywords:** chronic traumatic brain injury, rehabilitation, memory, ecological momentary intervention, text messaging, mobile health, mobile application, digital health, digital intervention

## Abstract

**Background:**

Memory and learning deficits are among the most impactful and longest-lasting symptoms experienced by people with chronic traumatic brain injury (TBI). Despite the persistence of post-TBI memory deficits and their implications for community reintegration, memory rehabilitation is restricted to short-term care within structured therapy sessions. Technology shows promise to extend memory rehabilitation into daily life and to increase the number and contextual diversity of learning opportunities. Ecological momentary assessment and intervention frameworks leverage mobile phone technology to assess and support individuals’ behaviors across contexts and have shown benefits in other chronic conditions. However, few studies have used regular outreach via text messaging for adults with chronic TBI, and none have done so to assess and support memory.

**Objective:**

This study aimed to develop and test the usability of memory ecological momentary intervention (MEMI), a text message–based assessment and intervention tool for memory in daily life. MEMI is designed to introduce new information, cue retrieval of the information, and assess learning across time and contexts. We tested MEMI via an iterative, user-centered design process to ready it for a future trial.

**Methods:**

We developed MEMI by leveraging automated text messages for prompts using a REDCap (Research Electronic Data Capture)/Twilio interface linking to the Gorilla web-based behavioral experimental platform. We recruited 14 adults with chronic, moderate-severe TBI from the Vanderbilt Brain Injury Patient Registry to participate in 3 rounds of usability testing: one round of ThinkAloud sessions using the platform and providing real-time feedback to an experimenter (n=4) and 2 rounds of real-world usability testing in which participants used MEMI in their daily lives for a week and provided feedback (n=5/round). We analyzed engagement and quantitative and qualitative user feedback to assess MEMI’s usability and acceptability.

**Results:**

Participants were highly engaged with MEMI, completing an average of 11.8 out of 12 (98%) possible sessions. They rated MEMI as highly usable, with scores on the System Usability Scale across all rounds equivalent to an A+ on a standardized scale. In semistructured interviews, they stated that MEMI was simple and easy to use, that daily retrieval sessions were not burdensome, and that they perceived MEMI as helpful for memory. We identified a few small issues (eg, instruction wording) and made improvements between usability testing rounds.

**Conclusions:**

Testing MEMI with adults with chronic TBI revealed that this technology is highly usable and favorably rated for this population. We incorporated feedback regarding users’ preferences and plan to test the efficacy of this tool in a future clinical trial.

## Introduction

### Overview

Every 21 seconds, one person in the United States sustains a traumatic brain injury (TBI) [1]. There has been a decrease in deaths caused by TBI in the last 20 years, but there has been no corresponding reduction in the rate of TBI-related disability [2]. TBI is increasingly recognized as a chronic condition, rather than an injury with finite recovery [3]. Symptoms, including memory and learning deficits, may persist for the rest of a patient’s life [3]. Yet, rehabilitation services are constrained to the acute and subacute phases of injury [4-6]. This leaves patients to cope with chronic disability over a lifetime without skilled support [4,7].

While the barriers to community reintegration after TBI are multifactorial [8], memory and the ability to (re)learn words and concepts are critical for rehabilitation potential and participation at school or work [9-12]. Memory disorders represent a key target for chronic care as they are among the most reported, costly, and lasting deficits after injury [9-11]. Memory deficits are present in more than half of patients with moderate-severe TBI [13] and can be detected in patients 10 years postinjury [10,14]. Yet, there has been limited progress in developing memory therapies for TBI in recent decades [15].

Learning is a complex and multifaceted phenomenon. Memory is not a process that occurs during a single event, but rather is an iterative process that repeats across multiple phases (encoding, consolidation, and retrieval) [16]. The act of memory retrieval is itself a form of learning that changes the nature and strength of that memory and its relations to other memories in the neocortex [17,18]. The memory literature distinguishes between short-term memory (in which a person remembers something when tested shortly after it has been encoded) and long-term memory (in which a person consolidates, or strengthens, a memory so that it is accessible and usable in flexible contexts long after it has been encoded) [16]. Existing clinical and research approaches that assess and treat memory in a single session are inadequate to capture and support the iterative process of encoding, consolidating, and retrieving a memory to build it into a long-term representation [12,19].

Multiple memory systems support dynamic elements of learning [20]. Considerable effort has been directed toward understanding the role of the declarative memory system in TBI outcomes. The declarative memory system depends critically on the hippocampus and medial temporal lobes [20], which are highly vulnerable to mechanisms of TBI [21-23]. This memory system binds arbitrary elements of an experience (eg, a word’s form to its meaning or a person’s name to their face) into lasting representations and facilitates the retrieval, recombination, and use of those representations in novel contexts [20]. Given the vulnerability of the declarative memory system in TBI, any memory assessment or treatment in TBI must consider the role of the declarative memory system in binding multiple elements of an experience to form a memory. Canonical neuropsychological assessments of memory for verbal material such as word lists, like the Rey Auditory Verbal Learning Test [24] and the California Verbal Learning Test [25], are less sensitive to the range and severity of deficits in declarative, or relational, memory that are common after TBI [26,27] and can be expanded upon by learning tasks that require individuals to form arbitrary relations between elements of an experience (eg, verbal and visual) and enact those relations over time and context.

Memory disorders should be assessed and treated in context [12,28]. Deficits that manifest in daily life may not be evident in a short session and controlled context, such as a medical visit, therapy session, or neuropsychological evaluation [4,29]. For example, recent work has shown that adults with chronic TBI do not strengthen (consolidate) their memories over time at the same rate as noninjured peers [19]. This deficit, which results in a growing learning gap, can only be identified by multiple memory assessments conducted throughout the learning process. Evidence in the cognitive neuroscience literature also indicates people with and those without brain damage benefit when the context of learning is diverse across time and space [30-32]. In memory rehabilitation, each retrieval of information is both an opportunity for assessment and (re)learning [16]. Contextual diversity includes retrieving information at dispersed times and in different locations (ie, spaced retrieval [33,34], mimicking the real-world nature of learning [31,32,34]. Yet, rehabilitation sessions often occur in a constrained context (ie, same room at the same time of day).

There is an opportunity to increase the contextual diversity of memory rehabilitation using technology. Ecological momentary assessment and intervention frameworks leverage mobile phone technology to assess and support individuals’ behaviors in real time, in their daily lives [35]. Technology has consistently shown benefits as an intervention tool in chronic conditions [36-39], and text messaging has been used to support daily behaviors like medication adherence in chronic conditions like diabetes [40,41]. Yet, few studies [42-48] have used regular outreach via text messaging for adults with chronic TBI, and none have used an ecological momentary intervention framework to assess and support memory [49].

### Objective

We developed memory ecological momentary intervention (MEMI) to address the dual goals of (1) assessing memory over time after TBI and (2) increasing the contextual diversity of retrieval opportunities to support memory for better long-term recall. We conducted 3 rounds of iterative usability testing with adults with chronic, moderate-severe TBI to identify and address any issues with functionality or acceptability before our evaluation of MEMI’s efficacy for assessing and supporting memory in a future trial.

## Methods

### Intervention Development

We developed a prototype for MEMI using simple text messaging with a mobile web interface. Text messages were delivered using Twilio integrated with REDCap (Research Electronic Data Capture) [50]. Participants clicked on a link in the text message to complete memory tasks on the Gorilla behavioral experiment platform [51]. We conducted internal testing with 8 members of our research team before moving MEMI to iterative usability testing as described below.

### Functionality

#### Overview

**Figure 1.**
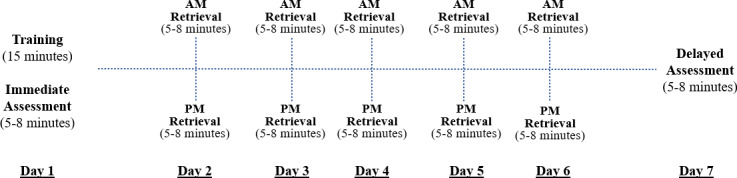
MEMI session schedule. Participants chose their morning and evening session times.

Participants received text message prompts to complete MEMI sessions throughout the week. The schedule was composed of 4 primary activities: training (1 time point; day 1), an immediate assessment (1 time point; day 1), AM/PM retrievals (10 time points; days 2‐6); and a delayed assessment (1 time point; day 7). This resulted in 12 possible sessions for each participant (1 session for training and immediate assessment, 10 retrieval sessions, and 1 session for delayed assessment). Figure 1 contains a visualization of the MEMI schedule. Participants selected the timing of their morning and evening text message prompts, and the experimenter entered these times into REDCap. Twilio then delivered the text message prompts at the chosen times. Each text message contained a link to a memory task on the Gorilla platform [51] and an approximate task duration (eg, “It is time to complete your memory task. This should take about 5 minutes;” Multimedia Appendix 1 contains a sample message). We describe the memory tasks below.

#### Context Checks

**Figure 2.**
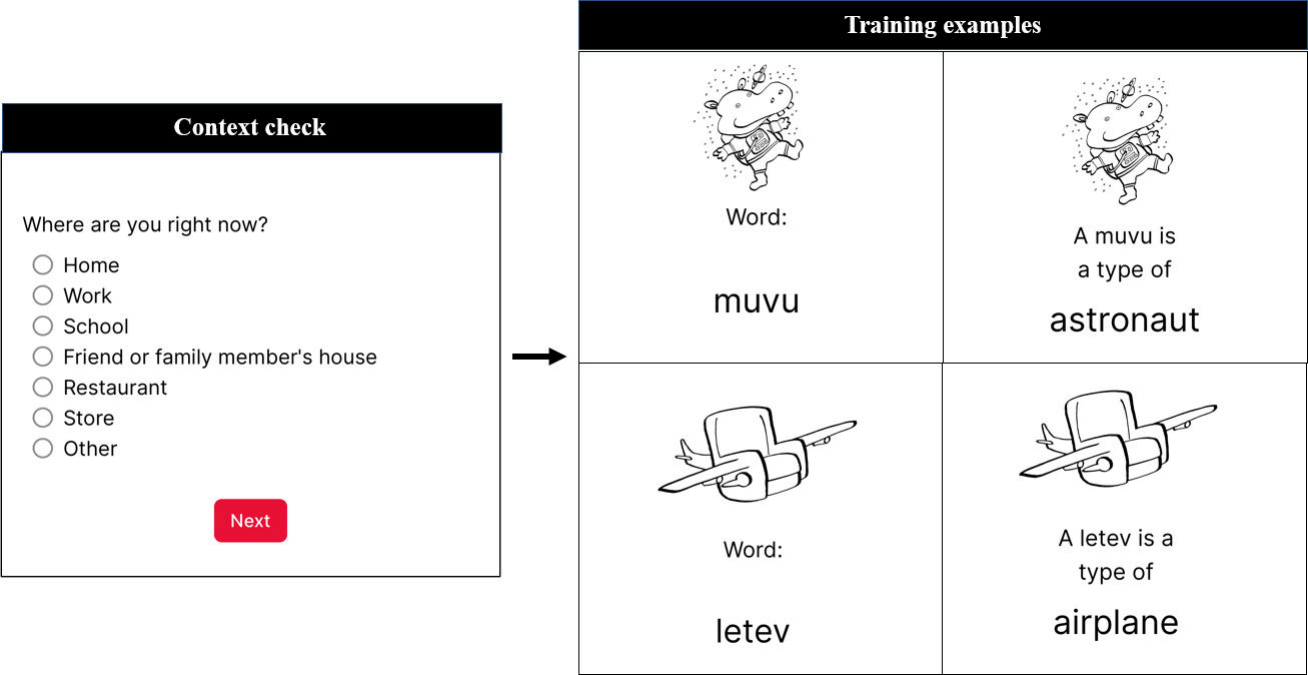
Sample context check and training items.

Before every MEMI session (training, retrieval, or assessment), we asked participants to indicate their spatial context (Figure 2). This will allow future assessment of the number of spatial contexts experienced by participants throughout the week, as well as whether training and assessment sessions were completed in the same spatial context.

#### Training

At the beginning of the week, participants completed a 15-minute training in which they saw each of 16 novel pieces of information (items) 4 times each. The items were presented via visual stimuli to ensure that all participants received the same exposure (eg, headphones were not required). The number of exposures was limited initially to keep the length of training and burden of using MEMI low. Figure 2 contains example images from MEMI training.

#### Immediate and Delayed Assessments

Immediately after the training, participants completed an assessment with several recall/recognition tasks designed to assess multiple levels of learning (immediate assessment). First, participants were asked to free type all the items that they learned (ie, free recall, the most robust indicator of learning [20]). Then, they responded to recall cues (eg, seeing an image and having to provide the name for that image). These tasks were designed to tap into memory for both individual elements of an experience (eg, a word form) and the relations between elements (eg, matching a word’s form to its meaning). The entire assessment took approximately 5‐8 minutes to complete. Participants completed the same assessment again on day 7 to assess their long-term retention of the trained information (delayed assessment). For both the immediate and delayed assessments, participants did not receive feedback on their responses. The goal of these sessions was to assess the memories that participants form immediately after training (encoding, immediate assessment) and how those memories changed over the course of the memory process (delayed assessment).

#### Retrieval Sessions

**Figure 3.**
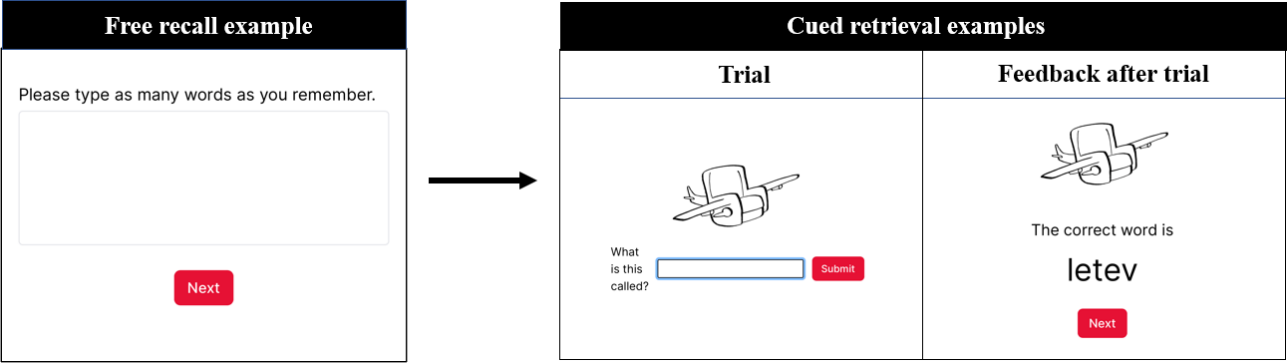
Sample free recall and cued retrieval prompts.

On days 2‐6, participants received text messages each morning and evening cueing them to complete short (5‐8 min) retrieval sessions on a subset of the trained items. Each retrieval session included 8 of the 16 trained items, and the items were cycled such that each item had the same number of retrieval opportunities over the course of the week. Participants completed a mix of the same free- and cued-recall tasks from the assessments. However, the retrieval sessions included an added component: after participants responded to each cued recall item, they saw the correct answer to ensure they gained exposure to the trained information. Notably, MEMI did not indicate if the participants’ responses were correct or incorrect to avoid corrective feedback that may discourage them from using the system. Figure 3 contains sample items from MEMI retrieval sessions.

### Content

We designed MEMI to flexibly deliver any target items, with the goal that this system will eventually be customizable to the information that people want to learn. To ensure that MEMI works across information types, we tested 2 types of items in usability testing. Half of the participants received novel word prompts like the ones in Figures2  3. The other half learned about names, occupations, and favorite colors associated with images of faces.

### Ethical Considerations

This work was approved by the Vanderbilt Human Research Program (Institutional Review Board #221667). Study procedures complied with the Helsinki Declaration. All participants gave written informed consent to participate in this study.

### Usability Testing

#### Sample and Recruitment

Participants were recruited from the Vanderbilt Brain Injury Patient Registry [52]. All participants in the Registry are in the chronic phase (>6 mo post injury) of moderate-severe TBI. We chose to focus on people with moderate-severe TBI as they are more likely to experience lasting memory deficits than people with mild TBI [10,13]. We chose to focus on the chronic phase of injury because we wanted to understand how people with chronic TBI use MEMI given our eventual goal to extend memory treatment to daily life and the chronic phase of injury. TBI severity was determined using the Mayo Classification Systemand [53] met at least one of these criteria: (1) Glasgow Coma Scale <13 within the first 24 hours of acute care admission, (2) positive neuroimaging finding (acute CT findings, or lesions visible on chronic MRI), (3) loss of consciousness >30 minutes, or (4) posttraumatic amnesia > 24 hours. Participants were 18 to 55 years of age; age was restricted to exclude developmental effects of TBI and conservatively limit the effects of age-related cognitive decline. They had no other neurological or cognitive disabilities aside from the qualifying brain injury. All participants had to own a mobile phone to participate in the intervention.

At the start of usability testing, we contacted a group of Registry participants that was selected to be diverse with regard to sex, age, and education to garner a wide range of perspectives on MEMI. We also intentionally recruited participants who may benefit from the integration of accessibility features within MEMI (eg, participants with hemiparesis who may use the text-to-speech tool). Each participant received an email and had the opportunity to respond to the email to indicate interest or schedule a phone call to learn more about the study. When a participant expressed interest, they were assigned to the round of usability testing that was ongoing at that time.

#### Procedures

##### Overview

We conducted usability testing across multiple, iterative rounds. We used 2 formats to address separate but complimentary usability evaluation goals [54,55]. The first testing round was in a ThinkAloud format [54-56] so that experimenters could observe participants’ use of MEMI and quickly address any usability challenges in an intensive and collaborative way. The subsequent testing rounds were in a real-world usability format so that participants could report on how MEMI worked for them across settings and in daily life.

##### ThinkAloud Usability Testing

In the first round of usability testing (n=4), participants used MEMI and verbally shared their thoughts on the system with an experimenter in real time. The experimenter could observe times they were unsure or struggled to complete a task. In addition, they were asked to provide feedback out loud (a method called ThinkAloud [54-56]) as they underwent the entire MEMI process (from receiving a text message to completing the memory tasks via the web) for a training, an assessment, and a single retrieval session. After completing the ThinkAloud session, participants responded to standardized feedback measures and completed a semistructured interview to assess usability and acceptability.

##### Real-World Usability Testing

Our next 2 rounds of usability testing (n=5/round) were conducted using a real-world format, so that participants used MEMI in their daily lives for a week and provided feedback on the experience. Participants met with an experimenter at the start of the week to provide consent and go through a handout describing key components of MEMI (Multimedia Appendix 1). Next, they used MEMI each day for a week (see Figure 1). Finally, they met with the experimenter again the day after they finished using MEMI to complete standardized feedback measures and a semistructured interview to assess usability and acceptability.

### Participant Characteristics Available From the Brain Injury Patient Registry

#### Demographic and Injury Characteristics

Demographic and injury characteristics were collected from available medical records and a semistructured participant interview when participants joined the Brain Injury Patient Registry. These included participant age, sex, years of education attainment, and time since injury, as well as acute injury characteristics used to categorize severity.

#### Memory

Participants in the Brain Injury Patient Registry also completed 2 neuropsychological assessments of episodic memory as part of their participation in the Registry: the Rey Auditory Verbal Learning Test [24] and the NIH Toolbox Picture Sequence Memory Test [57,58]. Multimedia Appendix 1 contains more information about the memory assessments.

### Measures Collected During Usability Testing

#### Digital Literacy

We assessed digital literacy using an adapted version of the Digital Health Literacy Scale, a validated 3-item measure of digital health care literacy [59]. Scores range from 0 to 12, with higher scores indicating better digital literacy. The original scale includes questions about using applications, setting up a video chat, and solving basic technical problems using a cell phone, computer, or another electronic device. Because our study focused on a mobile phone–based system, we modified the questions to focus on mobile phone use (Multimedia Appendix 1).

#### Engagement

We quantified the number and proportion of available sessions (out of a possible 12: 1 training session, 10 retrieval sessions, and 1 final memory test) completed by each participant in the real-world usability rounds. We did not prompt participants to complete sessions beyond the automated text messages they received. All participants were compensated the same amount, regardless of the number of sessions that they completed, so there was no financial incentive for higher engagement.

#### User Feedback

The same measures were used across all rounds of usability testing (ThinkAloud and real-world) to allow for direct comparison of results. We assessed usability using 10 items adapted from the System Usability Scale, a widely used standardized questionnaire for perceived usability [60,61]. Response options ranged from 1 (strongly disagree) to 5 (strongly agree) for each item. System Usability Scores range from 0 to 100, with higher scores indicating higher usability [62].

Following practical guidance [63], we modified some System Usability Scale items to make them more accessible for participants with TBI (Multimedia Appendix 1). For example, we changed “I found the system unnecessarily complex” to “I found the system too complicated.” We also added clarifying examples in some cases. For example, we changed “I found the various functions in this system were well integrated” to “I found that the parts of this system worked well together. For example, it was clear how to get to the memory questions from the text messages.”

We also asked 2 items to assess acceptability, which we reported separately: “using the system could be helpful for my memory” and “using the system was convenient.” Response options ranged from 1 (strongly disagree) to 5 (strongly agree).

After survey completion, we conducted a short semistructured interview with each participant to solicit additional feedback on usability and acceptability. We asked all participants what they liked best about MEMI, suggestions for improvement, if the scheduled text messages worked well in their daily lives, and whether the MEMI schedule was burdensome. We also asked each participant tailored questions based on the feedback they provided on the System Usability Scale and the acceptability items (eg, why they found the system useful for their memory).

### Analyses

We calculated descriptive statistics using R (version 4.2.1; The R Foundation). Interviews were audio-recorded, and key statements were transcribed. We undertook a pragmatic approach to analyze participant feedback quickly between rounds and change the intervention as needed in a timely fashion. Between rounds, author ELM identified actionable areas for improvement from participants’ feedback. Authors ELM and LSM decided if feedback should be incorporated before moving to the next round. Usability testing was completed after participants provided no more substantive feedback on MEMI, as 3 rounds of usability testing with 4 to 5 participants per round is considered sufficient to identify most usability problems [64,65].

## Results

### Participant Characteristics

#### Demographic and Injury Characteristics

Participants were on average 38.1 (SD 12.3, range 22-54) years old. Half of the sample was female. A total of 4 participants out of 14 (28.6%) had a high school education, 6 out of 14 (42.9%) had a college education, and 4 out of 14 (28.6%) had advanced degrees. Mean time since injury was 6.6 (SD 8.1, range 1‐23) years. Injury etiologies included motor vehicle accidents (8**/**14, 57.1%), falls (2**/**14, 14.2%), nonmotorized vehicle accidents (1**/**14, 7.1%), being hit by a car when walking (1**/**14, 7.1%), and one other etiology that is not nameable for participant confidentiality. One participant had hemiparesis affecting the ability to type. Table 1 separates participant demographic data by usability round. Multimedia Appendix 1 contains demographic and injury information about participants with TBI.

**Table 1.** Participant characteristics for the entire sample and per usability round.

Characteristics	Total (n=14)	Iterative testing round		
	ThinkAloud (n=4)	Real-world 1 (n=5)	Real-world 2 (n=5)
Age (in years), mean (SD)	38.1 (12.3)	40.5 (15.6)	42.0 (9.7)	32.4 (12.3)	
Sex (female), n (%)	7 (50%)	2 (50%)	2 (40%)	3 (60%)
Education (in years), mean (SD)	15.4 (2.7)	15.0 (2.0)	14.4 (3.6)	16.8 (2.3)
Months since injury, mean (SD)	79.6 (84.5)	100.8 (96.8)	101.0 (113.4)	41.4 (21.0)
Digital literacy, mean (SD)**^a^**	11.2 (1.1)	12.0 (0.0)	10.8 (1.3)	11.2 (1.3)

a Modified Digital Health Literacy Scale; scores range 0‐12, with higher scores indicating more digital literacy.

#### Memory

As a group, this sample exhibited deficits in episodic memory on neuropsychological testing. There was a range of memory abilities within the group. Multimedia Appendix 1 contains memory scores from neuropsychological testing.

#### Digital Health Literacy

The sample’s self-reported digital literacy using mobile phones was high, with an average score of 11.2/12 (SD 1.1, range 9‐12) on the mobile phone–focused Digital Health Literacy Scale.

### Usability Testing

Changing the target content presented between faces and words did not change user engagement or usability scores (Table 2), so we report results from all participants as a group below.

**Table 2.** Outcomes for the entire sample and per usability round.

Outcomes	Total (n=14)	Iterative testing round		
	ThinkAloud (n=4)	Real-world 1 (faces; n=5)	Real-world 2 (words; n=5)
Engagement, number of sessions completed (out of 12), mean (SD)	11.8 (0.4)	N/A^a^	11.6 (0.5)	12.0 (0.0)
Engagement, proportion of sessions completed, mean (SD)	98.3 (0.0)	N/A	96.7 (0.0)	100.0 (0.0)
System Usability Scale score, mean (SD)	91.4 (8.6)	95.0 (5.4)	87.5 (10.9)	93.8 (8.3)

a N/A: not applicable.

#### Engagement

Participants in both rounds of real-world usability testing exhibited high engagement with MEMI. As a group, participants completed an average of 11.8 (SD 0.4) out of 12 available sessions. All participants completed the training, immediate assessment, and delayed assessment; the limited missed sessions (3/120 total possible sessions) were midweek retrieval sessions. Table 2 contains engagement by round.

#### Usability and Acceptability

##### Quantitative Feedback

On the System Usability Scale, participants rated MEMI’s usability highly across all rounds. The average score across all rounds was 91.4 (SD 8.6), which is rated as an A+ score in the 96‐100 percentile range on a standardized scale [66]. Scores in each round (Table 2) qualified as A+scores. Every item was rated in the favorable range for all participants except two. On item 1 (“I would be open to using this system in the future”), 1 participant did not agree because they are trying to cut down on their mobile phone use. On item 7 (“I think that most people with traumatic brain injury would learn to use this system very quickly”), several participants stated that they were unsure because of the range of possible outcomes and ability levels after TBI. Otherwise, participants rated MEMI favorably across all components of usability: intuitive design (ie, understandability, clarity of instructions and tasks), subjective satisfaction (ie, enjoyment of use), and efficiency of use (ie, effectiveness in reaching desired goals).

Items added to assess acceptability indicated participants thought MEMI was convenient to use (average score 4.7/5, SD 0.6) and helpful for memory (average score 4.8/5, SD 0.4).

##### Qualitative Feedback

In semistructured follow-up interviews, several themes emerged from participant feedback. Below, we report common themes along with representative quotes from participants; participant information is reported in brackets (gender, age range, and usability testing round).

### Participants Found MEMI to Be Simple and Easy to Use

Consistent with scores on the System Usability Scale, participants noted that MEMI was easy to access and use. They noted that the consistency of the system allowed them to set expectations and complete the tasks.

*I thought it was really easy to use. It just had a really nice flow.*

[female, 26-30 y, real-world 2]

*As far as ease of use, it’s pretty much self-explanatory. If I can do it, anybody can do it.*

[female, 51-55 y, real-world 1]

*And the more simple something is, the less chance of something going wrong. It’s simple, but it accomplishes what it needs to accomplish.*

[female, 51-55 y, real-world 1]

### Participants Drew a Clear Distinction Between the Ease of Use and the Difficulty of the Memory Tasks

Several participants reported that the system was easy to use, but the memory tasks were very difficult.

*My answers weren’t confident, but I didn’t feel like it was a challenge to use it. Remembering all that was a challenge for the noggin!*

[male, 41-45 y old, real-world 1]

*I could use some tips on how to remember stuff, but the system was easy to use.*

[male, 41-45 y old, real-world 1]

### Participants Did Not Find MEMI Burdensome

No participants reported finding the MEMI schedule burdensome when asked directly. All participants said that the sessions were short, and several reported that they liked the predictability of the scheduled messages.

*I think after the first day and the second day, I got more used to the flow of it. I was even able to say, oh, and step outside, like at a friend’s house. And I know how long it takes me, so I feel more confident that I can open and finish it.*

[female, 26-30 y, real-world 2]

*I really liked it because it was very simple, and even on the days I couldn’t do it immediately, I knew I had something to do at 7AM and 7PM. And it was so simple because like, you just clicked on the link and knocked it out.*

[female, 19-25 y, real-world 2]

### Participants Felt MEMI Was Useful for Memory

All participants agreed that MEMI was useful for their memory. They identified 4 distinct reasons for this usefulness: noticing improvements in the memory task, tracking patterns in memory, the benefits of scheduled memory tasks, and diverse, cued repetitions. Table 3 contains sample quotes from participants in each of these areas.

**Table 3.** Participant feedback on the way memory ecological momentary intervention was useful for memory.

Theme	Sample quotes
Noticing improvement	*It was usable, and by the end of the time, I knew the words! So that felt really affirming.* [female, 26‐30 y, real-world 2] *I did terrible at the beginning, but by the end of it, I could remember most of the words.* [female, 51‐55 y, real-world 2] *I feel like I did get better, but sometimes through the weeks I slip anyways, and some of the times I didn’t do as well as I thought I should’ve. But then I’d do better the next time.* [female, 19‐25 y, real-world 2]
Tracking patterns in one’s own memory	*I liked it because the evening is when I’m most wore out. So even I could tell and see stuff myself, where I remembered it that morning but not that night.* [female, 19‐25 y, real-world 2] *I don’t know if it’s because I feel stressed right now, but I feel like I’ve been searching for more words the last couple of weeks. I think it’s because of what’s going on in my world at the moment. But this was definitely doable, which made me feel better. *[female, 51‐55 y, real-world 1]
Consistent, scheduled memory tasks each day	*Honestly, I did [like the scheduled messages] because it got me using my brain first thing in the morning. I normally let my mind chill in the morning, but it got me using my mind. *[male, 41‐45 y old, real-world 1] *I did like the constant and consistent reminder.* [male, 31‐35 y old, real-world 1]
Diverse, cued repetitions	*The way I perceived it, there were 3 parts to it. It was 3 different ways of emphasizing what something was. So you’re getting more per session than just repeating what things [are] over and over.* [female, 51‐55 y, real-world 1] *It reminded me of how my therapist would change things up on me. I think that helped.* [male, 41‐45 y old, real-world 1]

#### Participant Suggestions

We asked each participant for suggestions about how to improve MEMI. We incorporated 2 pieces of feedback after the ThinkAloud usability round: a request for a clear image indicating how to turn the phone to portrait mode to complete the memory tasks and a request for specific instructions on every item reminding participants to guess if they are unsure. We received positive feedback on each of these changes in subsequent rounds.

We also received feedback from some participants that we did not incorporate because it was only mentioned by a single participant and was not feasible in the current MEMI framework. One participant stated a preference to avoid technology: “I just feel like I am trying to, I don’t like phones. Like…I’m trying to use it less and less. Phones are amazing, they’re so powerful. But we need to not waste our lives there” [male, 21‐25 y old, real-world 2]. Another would have preferred to use MEMI on a desktop computer: “Probably most people would rather the cell phone. I even see people filling out applications on their cell phones. But for me, get to a computer” [male, 51‐55 y old, ThinkAloud]. Other participants requested changes to MEMI’s cueing structure based on personal preference, for example, a desire to slow down prompts, the opportunity to compare the correct answer to what they had written, or the option to use the words in a sentence.

#### Accessibility Modifications and MEMI

Two participants used MEMI in conjunction with other accessibility features that assist them with motor and cognitive deficits. One participant who found that typing is time-consuming due to hemiparesis used a text-to-speech feature to input their responses. This participant noted that they did have to correct occasional misspellings but otherwise found that the feature integrated well with MEMI since the tasks were in their phone’s web browser, although the tasks did take longer to complete. Another participant reported that they left MEMI text messages marked as unread until they completed the tasks, which is a tool that they use to accommodate for memory deficits in their daily correspondence.

## Discussion

### Principal Findings

Technology-based interventions provide an opportunity to extend the delivery of memory assessment and support for people with TBI. Text message–based ecological momentary intervention, specifically, may present a minimally burdensome way to extend memory rehabilitation to daily life. MEMI is the first such ecological momentary intervention for memory. We developed MEMI, a text message–based ecological momentary intervention system designed to assess and support memory using short sessions across time and context. We tested MEMI’s usability and acceptability among participants with TBI. Participants in all rounds of testing had favorable opinions of the system and responded frequently to the twice-daily memory task prompts.

Overall, participants were satisfied with MEMI and provided favorable ratings for its ease of use, intuitive design, enjoyment of use, and efficiency of use. The system provided consistent, structured memory support and allowed participants to monitor patterns in their own memory. Participants did not find twice-daily memory sessions to be burdensome and appreciated the opportunity to choose session times that were tailored to their schedules. Participants with accessibility challenges (eg, hemiparesis) were able to use MEMI with other accessibility features (eg, text-to-speech), which was possible because MEMI’s tasks take place on a phone’s integrated web browser.

We collected both quantitative and qualitative user feedback and system-collected engagement data to fully understand users’ experiences, improve functionality, and solve technical issues. Through these multiple data sources, we made improvements to MEMI and received unanticipated but actionable feedback on future research directions (eg, ways to change MEMI’s prompt levels that might enhance both memory and the user experience). This work highlights the need for participatory, user-centered design in all aspects of TBI rehabilitation research.

Usability testing is a necessary first step in user-centered intervention development, and this work suggests that MEMI may be usable for a wide range of people with chronic moderate-severe TBI. This study was conducted in a small sample of individuals with TBI, but we recruited the sample to be diverse with regard to sex, age, and education level. We also intentionally recruited participants who may benefit from integrating accessibility features with MEMI. There was also a range of memory abilities within this sample, and the sample exhibited deficits in episodic memory as a group on neuropsychological testing. Subsequent studies will include larger samples to understand MEMI’s acceptability and efficacy across the full range of heterogeneous post-TBI outcomes.

MEMI is distinct from existing models of memory rehabilitation (eg, weekly therapy sessions) because it leverages technology to deliver assessments and prompts repetitively in daily life, consistent with a context-sensitive rehabilitation approach [28]. It expands on existing technology-based memory interventions, which have largely been app-based and focused on using assistive technology to support prospective behaviors (eg, using a reminders app to take medications) [49,67-69]. We are aware of one study [70] in which participants received text messages containing target information (therapy goals) but were not asked to retrieve the information and respond themselves until tested at weekly intervals. By contrast, MEMI prompts retrieval to monitor and support an individual’s learning of specific information over time and across context. With its delivery of ecological momentary assessment and intervention via text messaging, MEMI may provide an approach to memory care that is more flexible and context-sensitive than traditional therapy but also more structured and informative about identifying specific learning patterns at the level of the individual than existing technology-delivered tools.

### Next Steps

MEMI was designed to extend the assessment and treatment of memory disorders beyond existing therapy contexts. This tool has multiple potential uses, including improving the assessment of the memory and learning processes in daily life for both clinical and research purposes. Using this tool in research may expand our understanding of how TBI affects memory over time and context, which can lead to new approaches to memory rehabilitation. Clinicians may program MEMI for patients to use between sessions to support memory for target items and to understand patterns in patients’ learning over time (eg, if disruptions occur in encoding, consolidation, or retrieval). Understanding learning patterns at the level of the individual will allow for a personalized medicine approach to memory treatment. After MEMI has undergone further testing to ensure its feasibility, acceptability, and efficacy, the system will be customizable to the information that people want to learn to increase the ecological validity of memory care.

Now that we have determined the usability of the tool, the next steps will be evaluating longer-term engagement, examining the feasibility of different prompt schedules and content, and then establishing efficacy in accordance with the ORBIT model of behavioral intervention development [71,72].

### Limitations

Although we did not specifically incentivize engagement with the study’s compensation structure, participants were compensated for providing feedback on their experience. Engagement with MEMI may be lower outside of a compensated research study or over a longer period of time. Furthermore, participants may have been reluctant to provide critical feedback due to compensation or their participation with the Brain Injury Patient Registry, although all participants were encouraged to provide their honest opinions. Some of our feedback items and interview questions did not have validity and reliability information to report, but composing our own items allowed us to target our research questions with face validity.

### Conclusions

Usability testing is a necessary first step in a participatory design process to ensure that effects identified in clinical trials are due to the intervention as intended and not impacted by difficulty with understanding or using the intervention. Because this is the first examination of an ecological momentary intervention for memory, it was critical to include people with a range of abilities in usability testing. Iterative usability testing of MEMI using multiple data sources revealed that MEMI is highly engaging and usable for people with TBI. Our findings emphasize the need for flexible, daily memory care in TBI and the importance of including people with TBI in a user-centered design process and using multiple sources of data to understand participant perspectives.

## Supplementary material

10.2196/59630 Multimedia Appendix 1 This includes all portions of the Multimedia Appendix referred to in the manuscript: (1) handout describing the memory ecological momentary intervention for participants, (2) participant and injury characteristics, (3) memory characteristics of the study sample, (4) modified System Usability Scale items, and (5) modified Digital Health Literacy Scale items.

## Abbreviations

MEMI: memory ecological momentary intervention
REDCap: Research Electronic Data Capture
TBI: traumatic brain injury
