# Supplementary material for: An Ecological Momentary Assessment and Intervention Tool for Memory in Chronic Traumatic Brain Injury: Development and Usability of Memory Ecological Momentary Intervention
Source: JMIR Rehabil Assist Technol. 2024 Nov 26;11:e59630. doi: 10.2196/59630 (PMC11612602; doi:10.2196/59630)
Supplement: Multimedia Appendix 1 [file rehab-v11-e59630-s001.pdf]

Appendix A: Handout describing MEMI for participants.

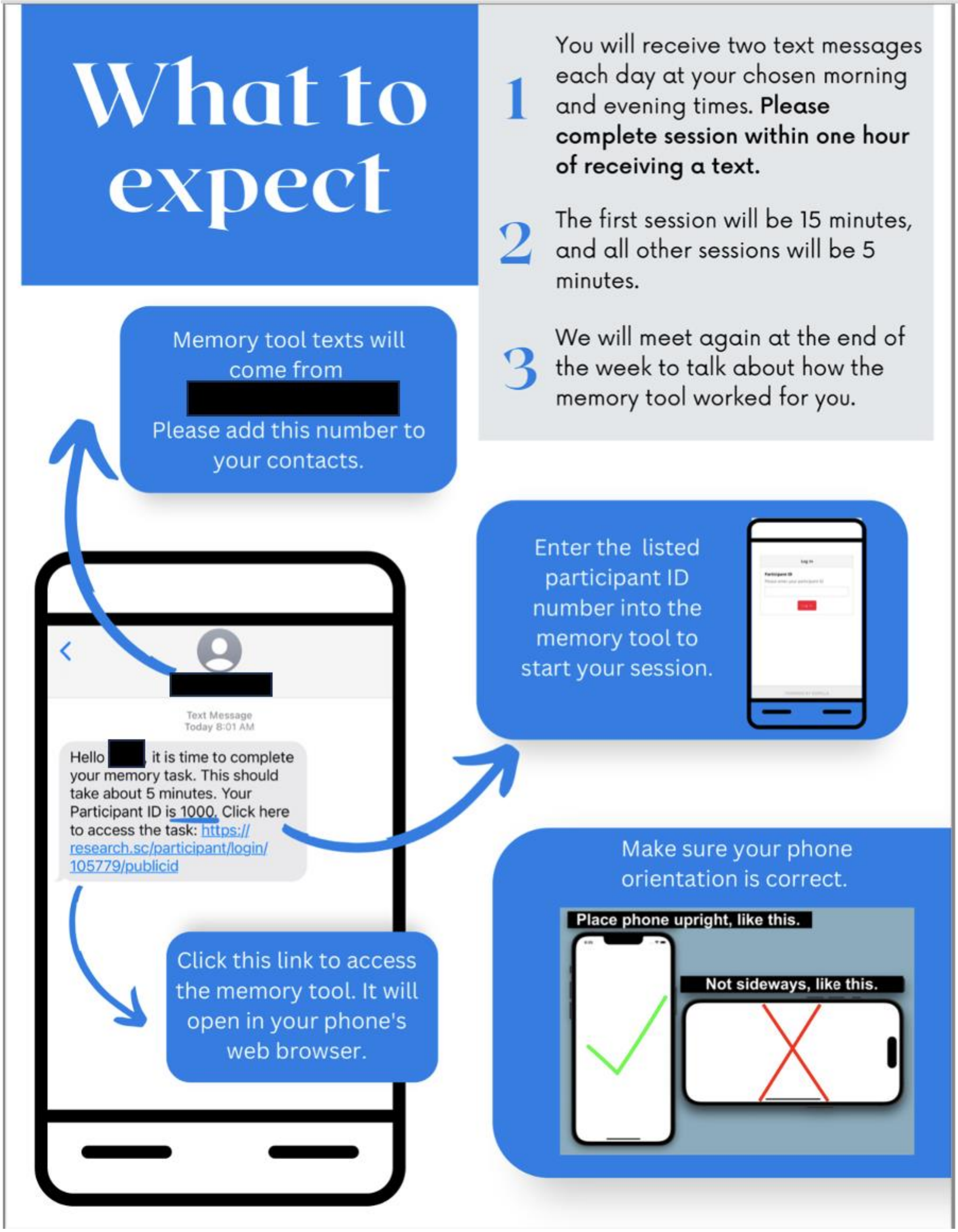

Appendix B: Participant and injury characteristics.

ID	Age	Edu	Etiology	TSO	GCS	LOC	PTA	Neuroimaging
5002	41-45	16	Non-motorized vehicle accident	278	3	>30 mins	>24 hrs	ICH
5003	26-30	18	Ped vs. auto	75	11	N/A	>24 hrs	SDH
5014	50-55	16	MVA	244	N/A	>30 mins	>24 hrs	N/A
5016	19-25	16	MVA	72	13	>30 mins	>24 hrs	SAH
5051	50-55	16	MVA	54	14	< 30 mins	< 24 hrs	SAH; SDH
5129	50-55	12	Other	36	12	< 30 mins	< 24 hrs	SDH; SAH
5141	26-30	12	MVA	33	13	>30 mins	<24 hrs	SDH
5145	31-35	20	MVA	152	12	>30 mins	>24 hrs	No
5149	19-25	14	MVA	33	3	< 30 mins	>24 hrs	IPH; SAH; DAI
5157	50-55	16	MCC	28	15	< 30 mins	No	SDH; SAH; IPH
5175	31-35	16	Ground-level fall	23	15	N/A	>24 hrs	SDH; SAH; Bifrontal Contusions
5178	31-35	12	MVA	15	3	>30 mins	>24 hrs	IPH; SAH; IVH; DAI
5180	41-45	12	MVA	24	3	>30 mins	>24 hrs	SAH; SDH; IPH
5187	21-25	16	Fall from height	48	N/A	>30 mins	>24 hrs	N/A

Note: ID = participant ID number. Age is provided in five-year ranges to protect participant confidentiality. Education (ed) reflects years of highest degree obtained. MVA = motor vehicle accident. MCC includes both motorcycle and snowmobile accidents. Non-motor = non-motorized vehicle accident. Ped vs. auto = participant was hit by car while walking or running. Time since onset (TSO) is presented in months. Glasgow Coma Scale (GCS) is total score. LOC = loss of consciousness. Mins= minutes. PTA = post-traumatic amnesia. Hrs = hours. N/A = data not available; No = no evidence of deficit on that measure. ICH = intracerebral hemorrhage. SDH = subdural hematoma. SAH = subarachnoid hemorrhage. EDH = epidural hematoma. IPH = intraparenchymal hemorrhage. IVH = intraventricular hemorrhage. DAI = diffuse axonal injury.

Appendix C: Memory characteristics of study sample.

Measures: Participants in the Brain Injury Patient Registry also completed two neuropsychological assessments of episodic memory as part of their participation in the Registry: the Rey Auditory Verbal Learning Test[1] and the NIH Toolbox Picture Sequence Memory Test[2,3]. On the Rey Auditory Verbal Learning Test, participants hear a list of 15 real words five times and attempt to free recall the words after each repetition. Their free recall for the 15 words is tested again after a 30 minute filled delay[1]. We use immediate (repetition of the target words after hearing the list once) and delayed (repetition of the target words after a 30 minute filled delay) recall to characterize this participant sample. The Picture Sequence Memory Test involves recalling series of illustrated activities, which increase in length as the subtest continues. Participants must recall the sequence of activities over two learning trials. Sequences vary in length from 6–18 pictures, depending on the participant's age, and participants receive credit for each adjacent pair of pictures they place correctly. The number of correct adjacent pairs is converted to a theta score, then a nationally normed standard score based on the participant's age[2,3].

Results: Data from the Rey Auditory Verbal Learning Test was available for 13/14 participants. On this assessment, participants remembered an average of 6.92/15 target words when tested immediately after hearing the word list once (SD: 2.9, range: 2-12). They remembered an average of 11/15 target words when tested after multiple repetitions of the word list and a 30-minute filled delay (SD: 4.0, range: 2-15). Data from the NIH Toolbox Picture Sequence Memory test was available for 9/14 participants. Average fully corrected t-score was 49.11 (SD: 4.7, range: 43-55), with an average percentile score of 50.1 (SD: 18.5, range: 24-79).

Appendix D: Modified System Usability Scale items.

1. I would be open to using the system in the future.
2. I found the system too complicated.
3. I thought the system was easy to use.
4. I think that I would need help to use this system.
5. I found that the parts of this system worked well together. For example, it was clear how to get to the memory questions from the text message.
6. I thought that the system did not work consistently enough. For example, the text messages did not always come at the correct times, or the link in the text message did not always take me to the same place.
7. I think that most people with traumatic brain injury would learn to use this system very quickly.
8. I found the system very clunky. That is, I found the system slow, awkward, or hard to use.
9. I felt very confident using the system.
10. I needed to learn a lot of things before I could use this system.
11. Using the system could be helpful for my memory.
12. Using the system was convenient.

Note: Items 1-10 were modified from the original System Usability Scale. Items 11-12 were added for the purposes of this project.

Appendix E: Modified Digital Health Literacy Scale items.

1. I can use applications (like Zoom, Instagram, or Messenger) on my cell phone on my own (without asking for help from someone else).
2. I can set up a video chat using my cell phone on my own (without asking for help from someone else).
3. I can solve basic technical issues on my cell phone on my own (without asking for help from someone else).

Appendix References

1. Schmidt M. *Rey Auditory Verbal Learning Test: A Handbook*. Western Psychological Services; 1996.
2. National Institutes of Health. NIH Toolbox Scoring and Interpretation Guide for the iPad. 2016. http://assistly-production.s3.amazonaws.com/228622/kb_article_attachments/98102/Toolbox_Scoring_and_Interpretation_Guide_for_iPad_v1.7_original.pdf?AWSAccessKeyId=AKIAJNSFWOZ6ZS23BMKQ&Expires=1581566214&Signature=ZBYA8k5wBvupD1KlTLgi0R9jquk%3D&response-co
3. Heaton RK, Akshoomoff N, Tulsky D, et al. Reliability and validity of composite scores from the NIH toolbox cognition battery in adults. *Journal of the International Neuropsychological Society*. 2014;20(6):588-598. doi:10.1017/S1355617714000241
